# Abdominal circumference but not the degree of lumbar flexion affects the accuracy of lumbar interspace identification by Tuffier’s line palpation method: an observational study

**DOI:** 10.1186/1471-2253-15-9

**Published:** 2015-01-21

**Authors:** Nan Lin, Yan Li, John F Bebawy, Jia Dong, Lin Hua

**Affiliations:** Department of Anesthesiology, Beijing Tiantan Hospital, Capital Medical University, Beijing, 100050 P.R. China; Northwestern University Feinberg School of Medicine, 251 E. Huron St., Suite F5-704, Chicago, IL 60611 USA; Biomedical Engineering Institute of Capital Medical University, Beijing, 100069 China

**Keywords:** Lumbar interspace, Spinal anesthesia, Cobb’s angle, Abdominal circumference

## Abstract

**Background:**

Lumbar puncture for spinal or epidural anesthesia is commonly performed by palpating bony landmarks, but identification of the desired intervertebral level is often inaccurate. It is unclear whether such inaccuracy is related to patient factors, such as body mass index and degree of lumbar flexion. We hypothesized that overweight patients and patients with less of an ability to hyperflex their lumbar spines are prone to inaccurate lumbar spinous intervertebral level identification.

**Methods:**

52 adult volunteers were included in this study. 7 anesthesiologists with different years of experience identified and marked subjects’ levels of the iliac crests, then marked the presumed interspaces. Lumbar X-ray was then performed with metal markers, and actual radiographic findings were identified and compared to the initial markings.

**Results:**

Patients with larger abdominal circumferences (mean (SD), 94.0(12.1) cm), higher body mass indices (25.9(3.9) kg/m^2^), and aged between 50 and 70 years old had lumbar interspaces that were higher than the presumed level; patients with smaller abdominal circumferences (82.8(13.5) cm) and lower body mass indices (21.6(4.1) kg/m^2^) had intervertebral levels that were lower than the presumed level. Cobb’s angle, indicating the degree of lumbar flexion, did not affect the accuracy obtained.

**Conclusions:**

Patients’ abdominal circumference, body mass index, and age are factors that may impact the accuracy of lumbar level identification. Tuffier’s line, as identified by palpation, does not seem to be a reliable landmark for proper lumbar interspace identification in all cases.

## Background

Lumbar puncture for spinal or epidural anesthesia or analgesia is commonly employed in routine clinical practice. The safety of subarachnoid puncture rests in accurately identifying the desired predetermined level for administering the anesthetic, so as to avoid the potentially disastrous complication of mechanical spinal cord injury [1, 2]. Spinal anesthesia is generally performed by the palpation of various bony landmarks, although this method yields accurate identification of the vertebral level in only 29% to 69% of cases [3–5]. Furthermore, this method is fraught with controversy as to the exact location of specific anatomical landmarks [4, 6]. The level of the iliac crests is the most popular anatomical reference landmark for estimating lumbar vertebral levels and is used most frequently in clinical practice. Tuffier’s line, which is the horizontal line connecting the highest points of the iliac crests, has been traditionally used to estimate the level of the L4 spinous process [7]. Anatomical landmark palpation, however, has been shown to be inaccurate, particularly in obese patients [4, 8]. Importantly, it is unclear whether being overweight itself hinders accurate palpation, or if lumbar spine hyperflexion, as affected by being overweight, yields this inaccuracy.

The purpose of the present study was to investigate whether subjects’ weight and degree of spine flexion affect the accuracy of lumbar interspace identification when using Tuffier’s line as an anatomical landmark. We hypothesized that overweight patients and a decreased capacity to hyperflex the lumbar spine are factors that contribute to the inaccuracy of lumbar interspace identification.

## Methods

Outpatients who were already prescribed a lumbar X-ray examination, and who did not possess serious lumbar disease, were approached to participate in this study. 52 volunteers gave written informed consent to be finally included in this study. All subjects were ≥18 years of age and able to cooperate with the investigator for anatomical examination and identification. Patients were excluded if unable to tolerate the lateral hyperflexion position due to local compression and bone pain, if they had severe spinal column or cord disease, if they had congenital or acquired spinal anomalies, or if pregnant. 7 anesthesiologists with different years of experience participated in evaluation independently as identifying practicioners to minimize observer bias. This was an observational study that was approved by the Institutional Review Board of Beijing Tiantan Hospital affiliated with Capital Medical University.

**Figure 1:**
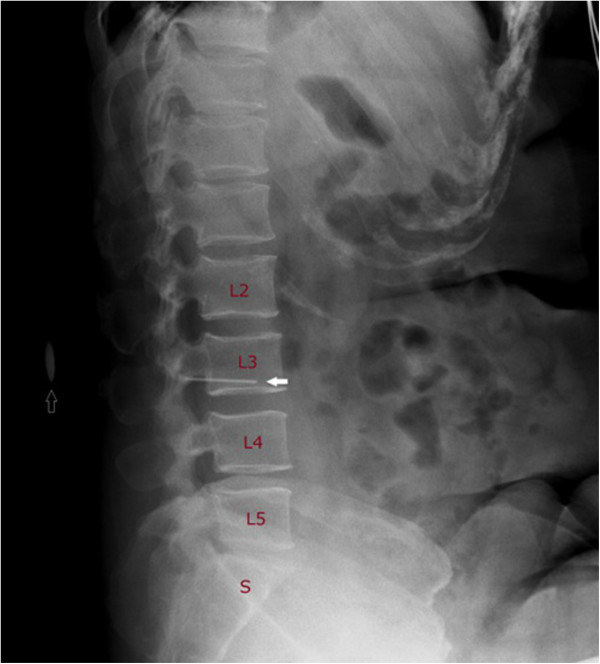
**A lumbar spine X-ray in the hyperflexion position.** The vertebrae from L2 to S are indicated. The solid arrow shows the radio-opaque marker where the highest point of the iliac crests was identified by palpation. The hollow arrow represents the spinous interspace identified by an anesthesiologist (the L2-L3 spinous process interspace in this film).

Subjects’ age, height, weight, abdominal circumference, and body mass index (BMI) were obtained before anatomical identification and lumbar X-ray examination. Anesthesiologists’ years of practice experience and operator hand dominance were also recorded. Subjects were first radiologically examined by means of an anterior-posterior lumbar spine X-ray in the supine position and lateral position, in order to obtain baseline images of the iliac bones and spinal column. They were then moved to the left lateral hyperflexion position and prepared for bony palpation and marking. For each subject, the anesthesiologists identified and marked the highest point of the iliac crests, then indicated the presumed L2-3 or L3-4 spinous process interspace in the left lateral hyperflexion position. A 5 cm long metal marker (radio-opaque) was then adhered to the identified iliac crests’ skin projection; a round metal button (diameter 1 cm) was adhered to mark the anesthesiologist’s chosen interspace. Hyperflexion lumbar spine X-ray was then performed with these metal (radio-opaque) markers in place (Figure 1). Finally, subjects’ actual radiographic findings (i.e., vertebral levels) were identified on the lateral hyperflexion X-ray image by another anesthesiologist who did not participate in the initial landmark identification. The palpating (identifying) anesthesiologists were not informed of the radiographic results.

### Tuffier’s line

From the “palpated” Tuffier’s line, a perpendicular line to the spinal column as seen on X-ray was identified to determine the corresponding vertebra or intervertebral space in both the supine and hyperflexion positions. In order to measure the distance between the “radiographic” Tuffier’s line and the presumed “palpated” Tuffier’s line, a perpendicular line was drawn from the radio-opaque (palpated) line to the center of the “double edge shadow” iliac crests’ line on the radiographs.

### Cobb angle

**Figure 2:**
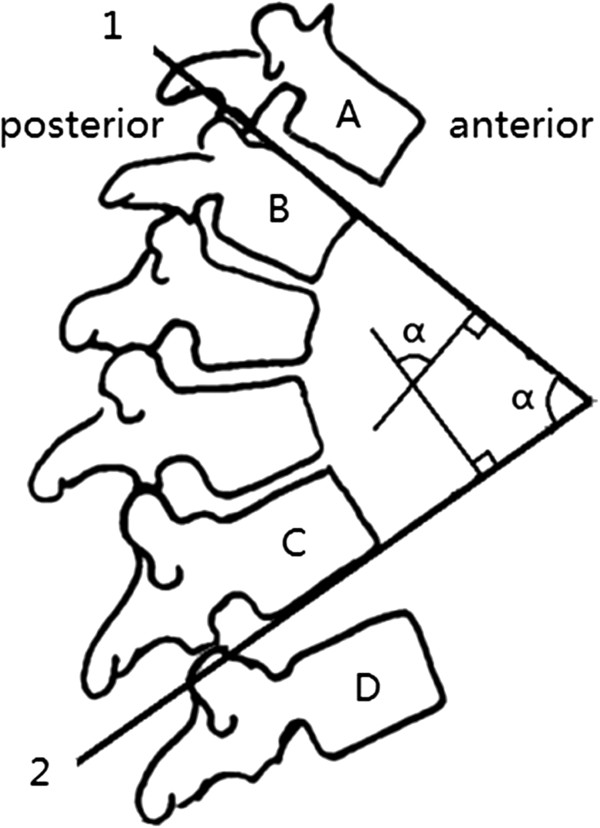
**Cobb Angle Calculation in the Sagittal Spine.** α represents the Cobb Angle. The curved segment has B as its top vertebra and C as its bottom vertebra. Vertebra B’s superior surface tilts to the side of the concavity of the curve, while vertebra A’s inferior surface tilts to the convexity side. The intervertebral space between vertebrae A and B on the side of the concavity is wider than the side of convexity. Vertebra C’s inferior surface tilts to the side of the concavity of the curve, while vertebra D starts to tilt to the convexity side; the intervertebral space between vertebrae C and D on the side of concavity is wider than the side of convexity. Line 1 is parallel to the superior surface of the top vertebra in the segmental curve (here vertebra B), while line 2 is parallel to the inferior surface of the bottom vertebra in the curve (here vertebra C). The angle formed by the intersection of lines 1 and 2 is the Cobb Angle, which is the “angle of the curve”.

The Cobb angle, used originally to measure the coronal degree of deformity in scoliosis patients, is still used today to measure spine curvature [9]. Cobb angle calculation was introduced in this study to evaluate subjects’ degree of hyperflexion. To measure the segmental Cobb angle, the angle formed between the intersecting lines projecting from the superior endplate of a cephalic vertebra and the inferior endplate of a caudal vertebra were used [10] (Figure 2). The Cobb angle was calculated in this study by computer analysis of the radiographs. Cobb angles were measured in both the lateral and lateral hyperflexion positions, and the difference between these two values was calculated.

### Statistical analysis

All statistical analyses were performed utilizing Statistical Package for the Social Sciences 17.0 (SPSS Inc., Chicago, IL). Kendall’s rank-correlation coefficient was used to assess the relationship between the accuracy obtained and subjects’ characteristics. Logistic regression was used to analyze the multiple possible factors associated with the accuracy obtained. Kolmogorov-Smirnov tests were used to describe the data distribution; the one-way ANOVA test was used for analyzing variance. Pearson correlation analysis was also used for evaluating the relationship between the Cobb’s angle difference and subjects’ characteristics. The Chi-square test was used to compare accuracies. Statistical significance was defined as a p value < 0.05. Based on preliminary data with an allowance for type I error of 0.05 and a power of 80%, we calculated that a sample size of 51 volunteers was needed for this study.

## Results

Volunteers were recruited for this study between August and October of 2011. One subject was withdrawn from the study because of back pain during palpation; three subjects’ radio-opaque markers were not visualized on the X-ray films and were excluded. Fifty-two patients were finally included in this study, with 7 “identifying” (palpating) anesthesiologists. Subjects’ demographic information (Table 1) revealed no difference in physical parameters between males and females.

**Table 1:** **Subjects’ demographic information**

	Male***(n = 17)***	Female***(n = 35)***	***P***value
Age (yrs)	46.9 (16.8)	48 (14.5)	0.516
Height (cm)	168.9 (7.2)	161.1 (5.8)	0.272
Weight (kg)	66.7 (13.1)	60.9 (9.9)	0.366
Abdominal Circumference (cm)	85.7 (11.6)	87.3 (11.8)	0.606
BMI (kg/m^2^)	23.4 (4.3)	23.4 (3.7)	0.621
ΔCobb Angle* (degrees)	15.9 (7.6)	11.8 (10.2)	0.152

Values are mean (SD).

*ΔCobb Angle represent the difference of Cobb angle between supine position and hyperflextion position.

For anesthesiologists with practice experiences < 3 years (2 practicioners identified 14 subjects), 3–5 years (1 practicioner identified 14 subjects), 6–10 years (2 practicioners identified 13 subjects) and > 10 years (2 practicioners identified 12 subjects), the accuracies in identifying the proper intervertebral space were 42.9%, 50%, 61.5%, and 72.7%, respectively. There was no statistical difference between groups (*P* = 0.459). One-way ANOVA revealed that subjects’ abdominal circumference (*P* = 0.506), BMI (*P* = 0.241) and age (*P* = 0.813) in each category of anesthesiologist experience had no statistical significance. It should be noted that this study was not powered sufficiently to identify a possible difference among anesthesiologists’ years of experience in terms of the accuracy they obtained. In the left lateral hyperflexion decubitus position, operator hand dominance did not affect the accuracy of identification (*P* = 0.657).

**Figure 3:**
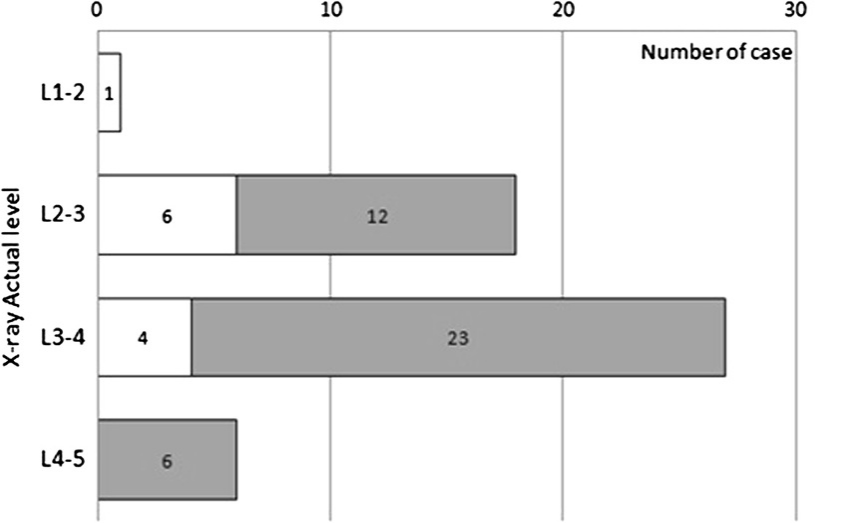
**Actual level under X-ray and palpation level by anesthesiologists.** The number of cases accumulated at each interspinous process level when anesthesiologists’ palpation aimed at assumed L2-L3 (□) or assumed L3-L4 (). The white bar represents the assumed level at L2-L3; the black bar represents the assumed level at L3-L4.

The overall accuracy of anesthesiologists in determining the actual intervertebral level was 55.8% (29 out of 52). Chi-square test showed no statistical difference in the accuracy obtained between the two genders (*P* = 0.378). Twenty five percent of patients (13 out of 52) demonstrated a higher actual interspace than the presumed interspace; 19.2% of patients (10 out of 52) demonstrated a lower radiographic interspace than presumed (Figure 3). Accuracy of lumbar level identification, as related to subjects’ BMI and degree of lumbar spine flexion, was the primary endpoint. There was an association between the accuracy of level identification and subjects’ abdominal circumference, BMI, and age (Table 2). In univariate analysis, patients with larger abdominal circumference, higher body mass index, and age between 50 and 70 years old had actual lumbar intervertebral levels that were higher than the presumed level; when all three variables were included in logistic regression, only age (*P* = 0.008) was a statistically significant predictor of identifying a higher level than presumed (for abdominal circumference, *P* = 0.731; for BMI > =25(n = 15) vs. BMI < 25(n = 37), *P* = 0.087). The cut-point used for BMI in this analysis was 25; the number of overweight (BMI = 25-29.9) patients in this population was 11, the number of grade 1 obesity (BMI = 30-34.9) patients was 4, and all others had a BMI less than 25. Patients with smaller abdominal circumference, lower body mass index, and younger patients had actual intervertebral levels that were lower than the presumed level. Differences in the Cobb angle between the lateral and hyperflexion positions, reflecting the degree of hyperflexion of the spine, did not affect the accuracy obtained (Table 2). However, less Cobb angle difference was correlated with higher abdominal circumference (Pearson Correlation = −0.438, *P* = 0.001) and middle age patients (50 – 70 years old) (Pearson Correlation = −0.297, *P* = 0.032). There was no significant statistical correlation between Cobb angle changes and height (Pearson Correlation = 0.205, *P* = 0.145), weight (Pearson Correlation = −0.150, *P* = 0.288), and BMI (Pearson Correlation = −0.262, *P* = 0.061).

**Table 2:** **Subjects’ characteristics in accurate and inaccurate spinous interspace identification**

	Actual interspace is one level lower than assumed	95% CI †	Actual interspace is the same as assumed	95% CI	Actual interspace is one level higher than assumed	95% CI	One-way ANOVA P value	Correlation analysis	
								Kendall’s tau-b(τ)	P value of the correlation
Height; cm	166.7 (8.3)	160.7,172.6	162.6 (7.4)	159.8,165.4	163.7 (5.8)	160.2,167.2	0.307	−0.070	0.535
Weight; kg	60.4 (13.1)	51.0,69.7	60.9 (9.6)	57.2,64.6	68.8 (11.8)	61.6,75.9	0.081	0.206	0.066
Abdominal Circumference; cm	82.8 (13.5)	73.1,92.4	85.0 (9.5)	81.3,88.6	94.0 (12.1)	86.6,101.3	0.029*	0.267	0.016**
BMI; kg/m^2^	21.6 (4.1)	18.7,24.6	22.9 (3.3)	21.7,24.2	25.9 (3.9)	23.5,28.2	0.015*	0.304	0.006**
Age; yrs	41.5 (15.8)	30.2,52.8	44.4 (14.6)	38.9,50.1	59.6 (9.0)	54.2,65.0	0.003*	0.342	0.002**
Cobb Angle in lateral position	2.9(0)	−0.6,6.4	6.2(7.5)	3.3,9.1	12.7(11.2)	5.9,19.4	0.016*	0.329	0.005**
Cobb Angle in hyperflexion position	17.5(8.7)	11.3,23.7	20.0(9.1)	16.5,23.4	23.5(15.1)	14.3,32.6	0.413	0.116	0.301
Cobb Angle between lateral and hyperflexion; degrees	14.6 (9.3)	7.9,21.3	13.7 (9.9)	10.0,17.5	10.8 (9.3)	5.2,16.4	0.573	−0.111	0.326
Distance from palpation point to iliac crest; mm	24.1 (18.6)	10.8,37.4	27.8 (20.7)	20.0,35.7	30.8 (19.1)	19.2,42.4	0.705	0.115	0.297

Values are mean (SD).

*P < 0.05 indicates a significant difference in one-way ANOVA that was used for comparing each cohort and analyzing variance, **P < 0.05 indicates the correlation is significant (2-tailed).† CI = Confidence Interval.

**Figure 4:**
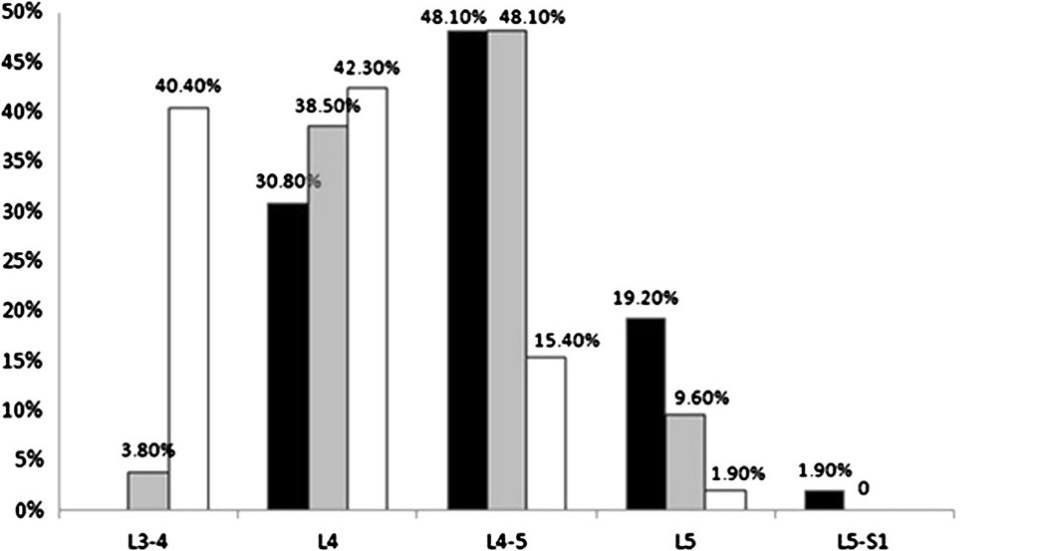
**Tuffier’s line intersecting with spine.** The distribution of Tuffier’s line intersecting with the spine in the supine position under X-ray (■), hyperflexion position under X-ray () and the estimated level under hyperflexion by palpation (□).

The radiographic level of Tuffier’s line in relation to the spine did not change significantly from the lateral to hyperflexion positions (Figure 4). The location of Tuffier’s line on X-ray correlated most closely with the L4-L5 interspace (48.1% for both supine and hyperflexion), while the “palpated” Tuffier’s line by anesthesiologists with subjects in hyperflexion correlated most closely with the L3-L4 interspace (40.4%) (Figure 4). There was a distance from the marked palpation level of Tuffier’s line to the radiographic iliac crests whether or not the intervertebral level was estimated accurately, although this was not statistically significant (Table 2).

## Discussion

The main findings of this study were that for subjects with larger abdominal circumference, higher BMI, and middle age (50 – 70 years old) patients, anesthesiologists tend to estimate the lumbar interspace at levels higher than the actual spinous process interspace. The degree of lumbar flexion is correlated with abdominal circumference and age, but does not seem to be associated with the accuracy obtained in and of itself.

Interestingly, we observed that the abdominal circumference of the males was smaller than that of the females in this population, which is different from what was observed in previous studies [11]. However, we found no statistical difference (P = 0.606) in our primary outcome between the genders. Perhaps we observed this distribution because no subgroup analysis of metabolic diseases (e.g., diabetes) was performed in our study; such analysis may affect gender distribution as it relates to abdominal circumference [12]. In other words, our study population, representing a mixture of diabetic and non-diabetic patients, may have contributed to the gender distribution we observed as it relates to abdominal circumference, which differs from other studies employing a larger sample size.

In this study, we used X-ray as the method of radiological evaluation of the actual anatomical location of physical landmarks. This was done, as opposed to using magnetic resonance imaging (MRI), because subjects were able to keep a hyperflexed position under X-ray, thus avoiding marker shift. All of the anesthesiologists in this study determined their chosen spinous process interspace by palpating Tuffier’s line. The anesthesiologists were asked to indicate the L2-3 or L3-4 interspace because these are the most common interspaces desired for spinal anesthesia.

Various patient factors resulted in the inaccuracy in identifying the correct lumbar interspace which we observed. Obviously, landmark depth increases with increasing subject BMI, which theoretically would influence palpation performance [13]. Although weight and BMI do not affect the radiographic Tuffier’s line [14], it would seem that palpated bony anatomy projections are affected by fat and muscle distribution. It appears that the palpation of bony landmarks in our study was affected by BMI and abdominal circumference, presumably because these landmarks are not always easily identified with abundant intervening tissue. In such cases, it may be difficult to accurately identify the level of the iliac crests, with the projection of these palpated landmarks tending to be higher than the actual Tuffier’s line. Lee *et al*. found that parturients with low trunk length/(abdominal circumference)^2^ values tended to have higher dermatomal levels during spinal anesthesia [15]. Therefore, a higher identified intervertebral level, combined with a higher dermatomal spread level of spinal anesthetic, could theoretically be dangerous in clinical practice and ought to be considered.

The lateral hyperflexion position is often used when performing spinal puncture and is thought to facilitate spinous process and interspace identification. Although we found that the degree of spine flexion was affected by abdominal circumferences and age, and also observed a difference in Cobb’s angle between the accurately and inaccurately identified groups in the neutral position, we did not observe a difference after full hyperflexion of the lumbar spine in the lateral position under X-ray just prior to anesthesiologist identification. Kim *et al*. found that the interspinous width of the L3-4 interspace increased almost one-fold with full flexion, but the position of the intercrestal line did not change [16], in agreement with our study. Thus, it appears that the flexion of the lumbar spine would facilitate needle insertion, but would not help (or impair) accurate level identification.

In our study, the radiographic Tuffier’s line was generally below the palpated line, and this distance between the actual iliac crests and their projection should always be considered when performing spinal anesthesia. In agreement with our study, the estimation of Tuffier’s line by palpation was not reliably accurate in a series of other studies [14, 17, 18]. The estimation of Tuffier’s line by palpation is very likely to be higher than its actual level. In this study, there was always a significant distance between the highest point of the iliac crests on X-ray and the palpated bony projections of the iliac crests among most of the subjects (50 out of 52, 96.2%). This discrepancy between palpation and “true anatomy” may predispose to spinal cord injuries with spinal anesthesia, as the distance between the palpated level of Tuffier’s line and the conus medullaris terminus may become much shorter, especially for older patients who already have shorter intervertebral distances [19]. We recommend that an “adjustment” of the palpated Tuffier’s line should be considered, with insertion of the spinal needle at a lower level or trajectory, to avoid potential contact with the conus medullaris terminus, especially in those middle age patients with increased BMI and larger abdominal circumference. For those patients whose actual interspaces were correct or lower than presumed, age, BMI, and abdominal circumference might partially predict the low risk of assuming too high an interspace. In any case, vigilance in all clinical scenarios is required.

It should be noted that anesthesiologists who participated this study located the spinous process interspaces only by Tuffier’s line, which may have limited their abilities to determine the interspace accurately. Furthermore, pelvic rotation may occur in the lateral position, which may have affected anesthesiologists’ estimation of the vertebral level, and we did not measure parameters of pelvic rotation on X-ray. Besides, all of the anesthesiologists who participated in this study were from one anesthesia department, and their practice may not reflect all practices. However, as described earlier, other investigations have also demonstrated a great deal of inaccuracy in assessing the proper intervertebral level by palpation, among both experienced and inexperienced practicioners [4, 6].

Lastly, and of note, this study was conducted in Beijing Tiantan Hospital, which is the general hospital in the capital of China and draws upon a huge immigrant population from all over China. Thus, subjects for this study should be largely representative of the Chinese population at large. We are hesitant, however, to generalize our findings to other populations in Asia, or to Western countries, as demographic characteristics may vary greatly from what we observed.

## Conclusions

Our study suggests that performing spinal anesthesia at the optimal interspace should be considered a procedure in which multiple considerations prevail, especially for patients with a larger abdominal circumference, a higher BMI, and middle aged patients; these patients are more likely to have their lumbar intervertebral levels be identified lower than their actual level. Hyperflexion of the lumbar spine did not affect the accuracy obtained in our study, so our initial hypothesis of insufficient lumbar hyperflexion relating to inaccuracy of level identification should be rejected. Accurate identification of lumbar intervertebral interspaces is paramount for the safety of spinal anesthesia, as many other studies have suggested [4, 19, 20], and the use of multiple bony landmarks rather than Tuffier’s line alone to identify the proper interspinous space, as well as choosing the L3-L4 interspace for spinal anesthesia, may provide more safety.

## Authors’ informations

Nan Lin and Yan Li have equal contribution to this study with designation of co-first author.

This study was carried out in the radiology center in Beijing Tiantan hospital.
